# Synergistic effect of indole‒3‒acetic acid and nitrogen on yield, sugar profile, and nitrogen utilization of salt-stressed sugar beet crop

**DOI:** 10.1186/s12870-025-06531-9

**Published:** 2025-05-13

**Authors:** Ahmed Shaaban, Hani S. Saudy, Mohamed A. M. Eid, Sammar F. Zahran, Ali A. A. Mekdad

**Affiliations:** 1 Agronomy Department, Faculty of Agriculture, Fayoum University, Fayoum, 63514 Egypt; 2 Agronomy Department, Faculty of Agriculture, Ain Shams University, Hadayek Shoubra, P.O. Box 68, Cairo, 11241 Egypt; 3 Soil, Water and Environment Research Institute, Agricultural Research Center, Giza, 12112 Egypt

**Keywords:** Auxin, Nitrogen supply, Non-sucrose impurities, Nutrient content, Salt stress, Sugar beet productivity

## Abstract

**Purpose:**

Salt stress often reduces plant efficiency in nutrient utilization, particularly nitrogen (N), leading to physiological disorders, primarily those related to phytohormones. Hence, the current study assessed the combined effect of indole-3-acetic acid (IAA) and N in inducing salt stress tolerance in sugar beet.

**Methods:**

Using a split-plot in randomized complete block design replicated thrice, the effect of three IAA levels (0, 150, and 300 mg L^− 1^, denoted IAA_0_, IAA_150_ and IAA_300_, respectively) and three N fertilization rates (240, 290, and 340 kg N ha^− 1^, abbreviated as N_240_, N_290_ and N_340_, respectively) on sugar beet’s growth, nutritional status, and quality and sugar quality in saline soil was explored.

**Results:**

Findings exhibited that IAA_300_ × N_340_ was the best combination for enhancing root diameter, leaf fresh weight, and leaf area index. Ionic homeostasis, expressed as the leaf K⁺/Na⁺ and Ca²⁺/Na⁺ ratios, reached its highest values with N_340_ (1.21 and 0.51, respectively), exceeding those observed with N_240_ and N_290_. The IAA_0_ or IAA_150_ × N_340_ gave the highest juice sodium content (34.0 and 33.8 mmol kg⁻¹, respectively), while N_240_ across all IAA treatments recorded the lowest ones. The IAA_300_ × N_340_ was the most effective practice for enhancing yields and N use efficiency in sugar beet, resulting in the highest root yield (97.6 t ha⁻¹), pure sugar yield (14.50 t ha⁻¹), and N use efficiency (0.342 kg root kg⁻¹ N), significantly outperforming other IAA × N interactions.

**Conclusion:**

In conclusion, progressive increases in IAA and N caused the enhancements sugar beet growth, yield, and related quality, since IAA at 300 mg L^− 1^ plus N at 340 kg N ha^− 1^ had the favorable synergism in this respect.

## Introduction

Marginal agricultural lands, particularly saline soils, often suffer from severe nutrient deficiencies, limiting crop growth and productivity [1, 2]. Various soil characteristics, including acidity, water status, nutrient cycling, and salinity, significantly affect nutrient bioavailability and homeostasis [3–5]. Among these factors, soil salinity is a critical threat to global food security, as it imposes abiotic stress on crops, leading to reduced final yield and quality of different crops [6, 7].

Nitrogen (N) as an essential macronutrient had the potential to alleviate the unfavorable impacts of abiotic stresses [8–10]. The N is significant mineral nutrient under abiotic stress situations, owing to the essentiality of organic nitrogenous complexes accumulation in plant [11]. In this respect, N is regarded as the basal constituent of all amino acids, proteins, and other nitrogenous osmolytes that assist in preserving plants from abiotic stress hazards [12]. Osmo-protectants related to N such as soluble proteins play a substantial influence in plant stresses tolerance. Proline amino acid acts as a macromolecular agent capable of safeguarding the integrity of protein that enhances the activities of different enzymes [13]. However, in addition to exclusive water uptake and ion toxicity, deficiency of nutrients such as N is one of the critical issues of soil salinity [14]. Accordingly, owing to the low availability of N in saline soils, impairment in plant growth and productivity is expected. Therefore, to save better plant growth in saline soils, exogenous supply of N via fertilization is recommended [15]. However, the excessive or insufficient N dramatically decreased the yield of sugar beet [16] and negatively affected sugar content and quality of roots [17, 18].

Plants respond to abiotic stress by accumulating osmoprotectants such as free amino acids and proline, which play a crucial role in stress tolerance and cellular homeostasis [19–21]. However, under such conditions, the endogenous levels of indole-3-acetic acid (IAA), a key phytohormone, tend to decline [22]. The IAA is a vital signaling molecule in higher plants, regulating various physiological processes, including cell elongation and overall plant growth [23]. Beyond its role in growth regulation, IAA also contributes to stress adaptation by enhancing plant resilience to environmental challenges [24]. Notably, studies suggest that the detrimental effects of nutritional stress on N metabolism can be alleviated through the exogenous IAA application, improving plant N use efficiency and stress tolerance [25]. In sugar beets, IAA has been reported to influence physiological traits and improve stress tolerance, including enhancing root growth and nutrient uptake efficiency under adverse conditions [22]. Additionally, exogenous IAA application has been linked to improved N metabolism, leading to enhanced yield and quality parameters in sugar beet cultivars [26].

However, available information regarding the synergistic effect between IAA and N on sugar beet physiology, yield and quality under salt stress is scarce. Therefore, the current research was performed to outstand the potential of IAA plus N in improving yield, sugar quality and N utilization of sugar beet grown in saline soil. This study hypothesizes that the artificial supply of IAA and N can restore the nutritional and physiological homeostasis of sugar beet subjected to salt stress.

## Materials and methods

### Experimental site attributes

**Fig. 1:**
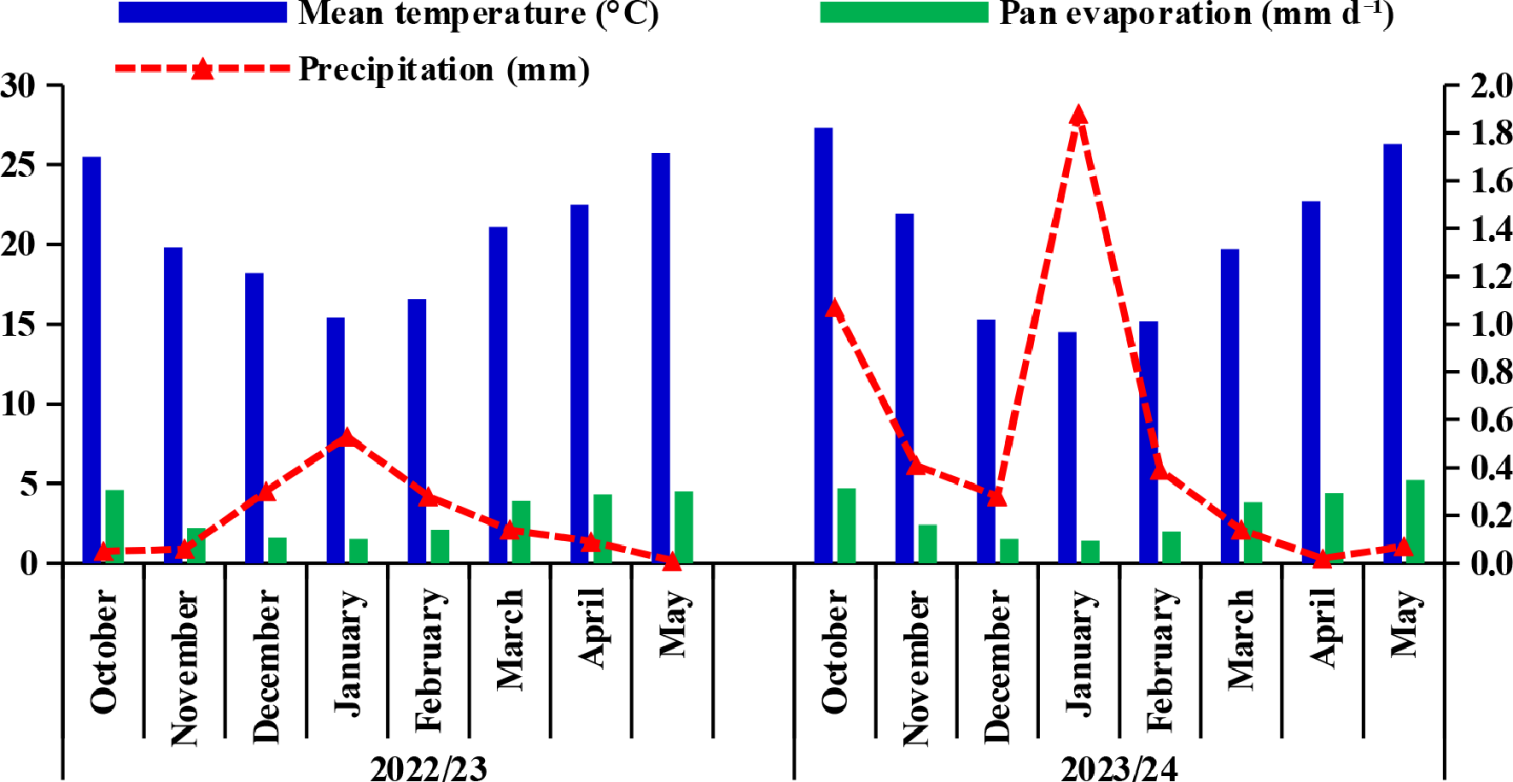
Monthly average temperature, pan evaporation, and precipitation of the experimental location during the sugar beet growing period for 2022/23 and 2023/24 cropping seasons

A field experiment was carried out during the 2022/23 and 2023/24 winter seasons at College of Agriculture Research Farm (latitudes 32° 42’ N and longitude: 29° 75’ E), Fayoum University, Fayoum, Egypt. The experimental soil is a saline loamy sand, classified by the Soil Survey Staff USDA as Typic Torripsamments, siliceous and hyperthermic. Representative soil samples were collected before planting in each season from the soil surface layer (taken from 0.0 to 0.5 m). The main physical and chemical properties of the soil and main climate features of the study area during the sugar beet (*Beta vulgaris* L.) growth period (from October to May) are illustrated in Table 1; Fig. 1, respectively.

**Table 1:** Main physical and chemical properties of the tested soil at a 0–50 cm depth before planting and and irrigation water (average for 2022/23 and 2023/24)

Property	Unit	Mean value ± SE	
		Soil	Irrigation water
Sand	(%)	71.6 ± 0.72	-
Silt		16.4 ± 0.49	-
Clay		12.0 ± 0.47	-
Soil texture		Loamy sand	-
Bulk density	(g cm^− 3^)	1.56 ± 0.06	-
Hydraulic conductivity	(cm^3^ h^− 1^)	2.41 ± 0.03	-
pH (1:2.5)		7.78 ± 0.11	7.49 ± 0.08
Saturated electrical conductivity	(dS m^− 1^)	6.98 ± 0.12	0.46 ± 0.05
Calcium carbonates	(%)	7.30 ± 0.23	-
Organic matter		1.12 ± 0.04	-
Available nitrogen	(mg kg^− 1^ soil)	54.32 ± 2.06	-
Available phosphorus		4.30 ± 0.23	-
Available potassium		43.12 ± 1.99	-
Available manganese		1.55 ± 0.03	-
Available iron		4.95 ± 0.05	-
Available zinc		0.72 ± 0.01	-
Available boron		0.35 ± 0.01	-

Each value represents mean of three replications ± standard error; SE

### Experimental design and sugar beet cultivation

The experiment was set up using a split-plot in randomized block design with three replications. Uniform and healthy multi-germ seeds of sugar beet (cv. BTS–301, Germany) were provided from the Sugar Crops Research Institute (SCRI), Agriculture Research Center, Egypt. The sugar beet seeds were sown on October 10, 2022/23, and October 12, 2023/24. Seeds were placed in drills on one ridge flank 0.2 m apart. Each experimental unit had 5 ridges, each 0.6 m and 3.5 m wide and length. Prior to sowing and while land was prepared, ordinary superphosphate, 110 kg P_2_O_5_ ha^− 1^, was added and mixed with soil. At 35 days from sowing (DFS), the excess sprouted plantlets were carefully reduced to retain one plant per drill, followed by top-dressing of potassium sulfate, 60 kg K_2_O ha^− 1^ and then undergo normal irrigation.

Treatments comprised three IAA (Sigma–Aldrich, USA, purity > 99.0%) concentrations at 0 (distilled water as a check; IAA_0_), 150 (IAA_150_), and 300 (IAA_300_) mg L^− 1^ were uniformly arranged in the main plots. Each IAA-applied concentration was sprayed thrice at 45, 60, and 75 days after planting (DAP) to a runoff early in the morning using a motorized pressurized backpack sprayer with a 20-L capacity. These ages align with specific sugar beet phenological development stages based on BBCH scale, a standardized codification system established by the Biologische Bundesanstalt, Bundessortenamt, and CHemische Industrie in Germany: BBCH 30/31 (10–20% ground coverage by leaves), BBCH 31/32 (20–30% ground coverage by leaves), and BBCH 32/33 (30–40% ground coverage by leaves) as reported by Meier [27]. Different IAA concentrations were prepared in distilled water, adding Tween^®^-20 (0.01%, *v/v*) as a surfactant agent to each treatment concentration to guarantee optimum penetration into leaf tissue. On basis the recommended N fertilization rate for sugar beet grown under newly reclaimed non-saline sandy soil conditions is 240 kg N ha^− 1^, three N fertilizer rates (i.e., 240 (N_240_), 290 (N_290_), and 340 (N_340_) kg ha^− 1^) as ammonium nitrate (33.5% N) were symmetrically top-dressed in the sub plots in three equal portions 30, 45, and 60 DFS. Due to the salt stress conditions in our study, we applied higher than recommended N rates (290 and 340 kg N ha^− 1^) based on the fact that N use efficiency is anticipated to decrease in saline soils. The traditional surface furrow irrigation system was used in the experimental field with irrigation freshwater having a 0.46 dS m^− 1^ salinity level. In this situation, the average amounts of irrigation water applied were 6316.9 m^3^ ha^− 1^ in the 2022/23 season, and 6190.2 m^3^ ha^− 1^ in the 2023/24 season, with following the local best practices for sugar beet cultivation under saline conditions in Egypt.

### Metrics

#### Growth attributes

At 95 DFS, the relative concentration of leaf chlorophyll (soil plant analysis development; SPAD_chlorophyll_ value) for six sugar beet plants, each with three completely expanded leaves, from each experimental plot was assesses via a SPAD-502 chlorophyll meter (Konica Minolta, Inc., Tokyo, Japan). Leaf area (cm² plant⁻¹) was measured for all leaves of each of the six selected sugar beet plants using a Planix 7 digital planometer (Tamaya Technics Inc., Tokyo, Japan). The leaf area index was determined by Eq. 1.

1 $$\:\text{L}\text{e}\text{a}\text{f}\:\text{a}\text{r}\text{e}\text{a}\:\text{i}\text{n}\text{d}\text{e}\text{x}=\left[\frac{\text{l}\text{e}\text{a}\text{f}\:\text{a}\text{r}\text{e}\text{a}\:\left({\text{c}\text{m}}^{2}{\text{p}\text{l}\text{a}\text{n}\text{t}}^{-1}\right)}{\text{g}\text{r}\text{o}\text{u}\text{n}\text{d}\:\text{a}\text{r}\text{e}\text{a}\:\left({\text{c}\text{m}}^{2}{\text{p}\text{l}\text{a}\text{n}\text{t}}^{-1}\right)}\right]$$

At root maturation (215 DFS), sugar beet plants were randomly sampling and gathered from each experimental plot. Further, the plant samples were submitted to the laboratory, and roots were washed. After that, root diameter was measured using a digital vernier caliper at the broadest root point. Also, the fresh weights of root and leaf per plant were recorded.

### Beet juice quality

Unbiased sample of six roots per treatment was taken to assess the juice quality traits at the Delta sugar beet factory located at Kafr El-Sheikh province, Egypt, as explained by McGinnis [28] and AOAC [29]. Sugar (i.e., polarity%) of beet roots was determined as described by McGinnis [28]. In this method, a clarified juice sample from the beet roots is prepared and then analyzed using a using ATAGO AP-300 digital automatic sugar polarimeter (Tokyo, Japan) to measure optical rotation, which is directly related to the sample’s sugar content (polarity). The soluble non-sucrose impurities, e.g., sodium (Na) and potassium (K) in mmol kg^− 1^ root were determined following a flame photometry method, while alpha-amino-N (α-AN) was measured using the ninhydrin and hydrindantin method [30]. The percentages of pure sugar (Eq. 2), loss sucrose (Eq. 3), and juice purity (Eq. 4) were calculated according to Harvey and Dotton [31] as follows:

2 $$\begin{aligned}\text{P}\text{u}\text{r}\text{e}\:\text{s}\text{u}\text{g}\text{a}\text{r}\:\left(\%\right)&=\text{s}\text{u}\text{g}\text{e}\text{r}\left(\%\right)\\&\quad-\left[\begin{aligned}&0.343\left(\text{K}+\text{N}\text{a}\right)\cr&\quad+0.094\:{\alpha\:}\cr&\quad-\text{A}\text{N}+0.29\end{aligned}\right]\end{aligned}$$

3 $$\:\text{L}\text{o}\text{s}\text{s}\:\text{s}\text{u}\text{c}\text{r}\text{o}\text{s}\text{e}\left(\% \right)=\text{s}\text{u}\text{g}\text{e}\text{r}\left(\%\right)-\text{p}\text{u}\text{r}\text{e}\:\text{s}\text{u}\text{g}\text{a}\text{r}\left(\% \right)$$

4 $$\:\text{J}\text{u}\text{i}\text{c}\text{e}\:\text{p}\text{u}\text{r}\text{i}\text{t}\text{y}\left(\% \right)=\left[\frac{\text{p}\text{u}\text{r}\text{e}\:\text{s}\text{u}\text{g}\text{e}\text{r}\left( \% \right)}{\text{s}\text{u}\text{g}\text{e}\text{r}\left(\% \right)}\right]\times\:100$$

### Mineral content in sugar beet leaves

After being ground, a 200 mg dried powder sample of sugar beet leaves (taken at 95 DFS) was digested with 10 mL H₂SO₄-HClO4 mixture (3:1, *v/v*) in a digestive vessel by heating up to 300 ºC until the digestion mixture was colorless. Each sample was diluted to 100 ml with distilled water in a volumetric flask. Using Gerhardt’s micro Kjeldahl device, the total N concentration in mg g^− 1^ leaf dry weight (LDW) was determined following the Kjeldahl procedure [29]. The total phosphorus (P) concentration (mg g^− 1^ LDW) was determined following the standard molybdenum blue spectrophotometric procedure [32]. The total concentration of cationic elements (i.e., potassium; K^+^ sodium; Na^+^, and calcium; Ca^2+^ in mg g^− 1^ LDW) in sugar beet leaves was determined using an Agilent Spectra-55 AA flame atomic absorption spectroscopy (CA, USA) according to Johnson’s [33] method.

### Sugar beet yields and nitrogen-use efficiency

Sugar beet plants from each experimental plot were harvested, cleaned, and topped. The weight of their roots, along with the root weight of ten plants sampled earlier, was measured and then converted to root yield ha^− 1^. Pure sugar yield ha^− 1^ was estimated by multiplying root yield by pure sugar content. The N use efficiency based on root yield [R-NUE = root yield (kg)/total N fertilizer applied (kg)] was calculated (kg roots kg N⁻¹) according to Moll et al. [34].

### Statistical analysis

Once the homogeneity of variances (for the data of both seasons) via Levene’s test (*p* > 0.05) was verified, the dataset for each tested variable/attribute met this statistical assumption, justifying a combined ANOVA). Herein, the data were statistically analyzed by Genstat software package (VSN International Ltd., Oxford, UK). For separating means, Duncan’s multiple range test was used at *p* ˂ 0.05 and *p* ˂ 0.01 levels of probability. Furthermore, the R (version 4.0.2) and IBM SPSS (version 25, SPSS Inc., Chicago, IL, USA) statistical software programs were utilized for the PCA biplot-building, Pearson’s correlation heat-map, and automatic linear modeling.

## Result

### Sugar beet growth

The differences in all studied growth traits of sugar beet due to application of IAA and N and their interaction were significant (*p* ˂ 0.05), except root fresh weight and SPAD_chlorophyll_ value with the interaction (Table 2). Progressive increase in IAA dose showed increases in the growth traits. Thus, IAA_300_ was the effective practice recording 5.3, 15.9, 29.5, 5.2, and 22.1% increases in root diameter, root fresh weight, leaf fresh weight, SPAD_chlorophyll_ value and leaf area index, respectively, compared to IAA_0_ (control treatment). The highest application rate of N, i.e., 340 kg N ha^− 1^ (N_340_) possessed the maximum increases in all sugar beet growth traits surpassing (*p* ˂ 0.05) the lower N rates (N_240_ and N_290_) by approximately 16.6 and 9.0%, 45.6 and 17.5%, 78.0 and 34.8%, 16.2 and 6.7%, and 87.3 and 45.9% for root diameter, root fresh weight, leaf fresh weight, SPAD_chlorophyll_ value and leaf area index, respectively. The interaction effect between IAA and N rate revealed that IAA_300_ × N_340_ was the best combination for enhancing root diameter, leaf fresh weight, and leaf area index. Under different IAA levels, the highest N rate showed remarkable values greater than the lower N rate.

**Table 2:** Root diameter, root fresh weight, leaf fresh weight, relative chlorophyll content (SPAD_chlorophyll_ value), and leaf area index of sugar beet plant as influenced by indole‒3‒acetic acid (IAA) and nitrogen (N) rate (data average for 2022/23 and 2023/24 cropping seasons)

Treatment		Root diameter (cm)	Root fresh weight	Leaf fresh weight	SPAD_chlorophyll_ value	Leaf area index
			(kg plant^− 1^)			
IAA (mg L^− 1^)						
IAA_0_		12.82 ± 0.29c	1.57 ± 0.07c	0.61 ± 0.03c	53.4 ± 1.6c	4.56 ± 0.28c
IAA_150_		13.14 ± 0.29b	1.70 ± 0.07b	0.66 ± 0.04b	54.7 ± 1.6b	4.97 ± 0.33b
IAA_300_		13.50 ± 0.32a	1.82 ± 0.07a	0.79 ± 0.06a	56.2 ± 1.6a	5.57 ± 0.40a
N rate (kg ha^− 1^)						
N_240_		12.19 ± 1.24c	1.38 ± 0.03c	0.50 ± 0.01c	50.5 ± 1.3c	3.63 ± 0.11c
N_290_		13.04 ± 0.24b	1.71 ± 0.03b	0.66 ± 0.02b	55.0 ± 1.5b	4.66 ± 0.12b
N_340_		14.22 ± 0.22a	2.01 ± 0.05a	0.89 ± 0.04a	58.7 ± 1.5a	6.80 ± 0.21a
IAA × N rate						
IAA_0_	N_240_	11.94 ± 0.44 g	1.22 ± 0.04a	0.45 ± 0.02 g	48.9 ± 2.4a	3.36 ± 0.17 g
	N_290_	12.80 ± 0.45e	1.61 ± 0.03a	0.62 ± 0.03ef	53.8 ± 2.5a	4.31 ± 0.16e
	N_340_	13.71 ± 0.35c	1.90 ± 0.05a	0.77 ± 0.04bc	57.4 ± 2.7a	6.00 ± 0.17c
IAA_150_	N_240_	12.20 ± 0.42 fg	1.39 ± 0.02a	0.49 ± 0.01 g	50.6 ± 2.3a	3.61 ± 0.19 g
	N_290_	13.05 ± 0.43de	1.71 ± 0.04a	0.67 ± 0.03de	55.0 ± 2.7a	4.55 ± 0.18e
	N_340_	14.15 ± 0.36b	2.00 ± 0.06a	0.81 ± 0.04b	58.5 ± 2.8a	6.73 ± 0.23b
IAA_300_	N_240_	12.43 ± 0.46f	1.52 ± 0.02a	0.56 ± 0.02f	52.1 ± 2.3a	3.92 ± 0.17f
	N_290_	13.27 ± 0.39d	1.80 ± 0.05a	0.71 ± 0.03 cd	56.2 ± 2.8a	5.11 ± 0.16d
	N_340_	14.81 ± 0.34a	2.14 ± 0.09a	1.10 ± 0.06a	60.2 ± 2.9a	7.67 ± 0.29a
*p*-value						
IAA		< 0.001^**^	< 0.001^**^	< 0.001^**^	< 0.001^**^	< 0.001^**^
N rate		0.031^*^	< 0.001^**^	< 0.001^**^	< 0.001^**^	< 0.001^**^
IAA × N rate		0.006^**^	0.670^ns^	< 0.001^**^	0.964^ns^	< 0.001^**^
CV (%)		1.2	1.5	5.5	2.0	3.4

IAA_0_, IAA_150_ and IAA_300_: spraying of indole‒3‒acetic acid at rates of 0, 150 and 300 mg L^− 1^, respectively; N_240_, N_290_ and N_340_: soil application of nitrogen at rates of 240, 290 and 340 kg N ha^− 1^, respectively. Each value in the table represents mean of three replications ± SE. * and ** indicate significance levels at *p* ˂ 0.05 and *p* ˂ 0.01, respectively; ns denotes no significance. According to Duncan’s multiple range test at *p* ˂ 0.05 different letters indicate significant differences between mean values for each factor in each column. CV: coefficient of variation.

### Mineral content in sugar beet leaves

As shown in Table 3, leaf mineral contents and ionic homeostasis significantly (*p* ˂ 0.05) influenced by the main effect of IAA and N rate, while the interaction was not significant. Concerning IAA, IAA_300_ recorded the maximum values of leaf mineral contents and ionic homeostasis surpassing the other IAA levels, except IAA_150_ for leaf P content. The increases in leaf N content, leaf P content, leaf K^+^ content, leaf K^+^/Na^+^ ratio and leaf Ca^2+^/Na^+^ ratio due to IAA_300_ application amounted to 10.0, 10.9, 2.1, 6.9 and 10.5%, respectively compared to the control treatment.

**Table 3:** Leaf mineral (nitrogen; N, phosphorus; P, and potassium; K^+^) contents and ionic homeostasis (K^+^/Na^+^ and Ca^2+^/Na^+^ ratios) of sugar beet plant as influenced by indole‒3‒acetic acid (IAA) and nitrogen (N) rate (data average for 2022/23 and 2023/24 cropping seasons)

Treatment		Leaf *N*	Leaf *P*	Leaf K^+^	Leaf K^+^/Na^+^	Leaf Ca^2+^/Na^+^	
		(mg g^− 1^ leaf dry weight)					
IAA (mg L^− 1^)							
IAA_0_		21.7 ± 1.0c	2.74 ± 0.14b	28.8 ± 0.80c	1.01 ± 0.05b	0.38 ± 0.03b	
IAA_150_		22.3 ± 1.1b	2.97 ± 0.14a	29.2 ± 0.79b	1.02 ± 0.05b	0.39 ± 0.03b	
IAA_300_		24.3 ± 1.2a	3.04 ± 0.16a	29.4 ± 0.84a	1.08 ± 0.06a	0.42 ± 0.03a	
N rate (kg ha^− 1^)							
N_240_		19.3 ± 1.3b	2.39 ± 0.16b	26.1 ± 0.85b	0.86 ± 0.03c	0.30 ± 0.01c	
N_290_		23.2 ± 0.5a	3.08 ± 0.07a	29.9 ± 0.36a	1.03 ± 0.03b	0.38 ± 0.01b	
N_340_		25.8 ± 0.7a	3.28 ± 0.1a	31.3 ± 0.55a	1.21 ± 0.04a	0.51 ± 0.02a	
IAA × N rate							
IAA_0_	N_240_	18.5 ± 2.3a	2.26 ± 0.28a	25.8 ± 1.52a	0.84 ± 0.06a	0.29 ± 0.02a	
	N_290_	22.0 ± 0.6a	2.90 ± 0.11a	29.7 ± 0.64a	1.02 ± 0.06a	0.36 ± 0.02a	
	N_340_	24.5 ± 1.0a	3.05 ± 0.16a	30.9 ± 0.97a	1.16 ± 0.07a	0.48 ± 0.05a	
IAA_150_	N_240_	19.0 ± 2.4a	2.45 ± 0.30a	26.2 ± 1.55a	0.85 ± 0.07a	0.30 ± 0.03a	
	N_290_	22.8 ± 0.7a	3.16 ± 0.10a	30.0 ± 0.64a	1.03 ± 0.06a	0.37 ± 0.02a	
	N_340_	25.3 ± 1.2a	3.31 ± 0.16a	31.4 ± 1.01a	1.18 ± 0.07a	0.49 ± 0.05a	
IAA_300_	N_240_	20.5 ± 2.6a	2.46 ± 0.32a	26.3 ± 1.59a	0.87 ± 0.06a	0.32 ± 0.02a	
	N_290_	24.8 ± 0.8a	3.19 ± 0.14a	30.1 ± 0.67a	1.06 ± 0.07a	0.40 ± 0.03a	
	N_340_	25.7 ± 1.3a	3.48 ± 0.20a	31.7 ± 1.02a	1.30 ± 0.08a	0.55 ± 0.04a	
*p*-value							
IAA		< 0.001^**^	< 0.001^**^	< 0.001^**^	0.044^*^	< 0.001^**^	
N rate		< 0.001^**^	< 0.001^**^	< 0.001^**^	< 0.001^**^	< 0.001^**^	
IAA × N rate		0.997^ns^	0.910^ns^	1.000^ns^	0.779^ns^	0.862^ns^	
CV (%)		10.8	6.1	6.0	7.7	9.2	

IAA_0_, IAA_150_ and IAA_300_: spraying of indole‒3‒acetic acid at rates of 0, 150 and 300 mg L^− 1^, respectively; N_240_, N_290_ and N_340_: soil application of nitrogen at rates of 240, 290 and 340 kg N ha^− 1^, respectively. N: nitrogen, P: phosphorus, K^+^: potassium, Na^+^: sodium, and Ca^2+^: calcium. Each value in the table represents mean of three replications ± SE. * and ** indicate significance levels at *p* ˂ 0.05 and *p* ˂ 0.01, respectively; ns denotes no significance. According to Duncan’s multiple range test at *p* ˂ 0.05 different letters indicate significant differences between mean values for each factor in each column. CV: coefficient of variation.

Application of N_340_ or N_290_ possessed the highest leaf mineral contents exceeding application of N_240_ by about 1.33 and 1.20 times for leaf N content; 1.37 and 1.28 for leaf P content and 1.20 and 1.14 for leaf K^+^ content, respectively. Ionic homeostasis expressed in leaf K^+^/Na^+^ ratio and leaf Ca^2+^/Na^+^ ratio exhibited the maximum values with N_340_ higher than each of N_240_ and N_290_.

### Sugar content and juice non-sucrose impurities

By supplying sugar beet plants with IAA_300_, the maximum values of sugar content and non-sucrose impurities were recorded (Table 4). However, the differences among IAA_0_, IAA_150_ or AA_300_ for Na content and α-AN content were not significant (*p* > 0.05). The greatest values of sugar content and K content (with N_240_) as well as Na content α-AN content (with N_340_) were observed. Among the non-sucrose impurities, only Na content significantly (*p* ˂ 0.05) affected by the interaction between IAA and N rate. IAA_0_ or IAA_150_ × N_340_ gave the maximum values, while all IAA levels × IAA_150_ exhibited the lowest ones.

**Table 4:** Sugar content and juice non-sucrose impurities (sodium; Na, potassium; K, and alpha-amino-nitrogen; α-AN) of sugar beet as influenced by indole‒3‒acetic acid (IAA) and nitrogen (N) rate (data average for 2022/23 and 2023/24 cropping seasons)

Treatment		Sugar content (%)	Juice Na	Juice K	Juice α-AN
			(mmol kg^− 1^ root)		
IAA (mg L^− 1^)					
IAA_0_		18.5 ± 0.36c	30.3 ± 0.84a	33.4 ± 0.77b	11.0 ± 0.38a
IAA_150_		18.8 ± 0.38b	30.1 ± 0.89a	33.6 ± 0.66b	10.99 ± 0.33a
IAA_300_		19.0 ± 0.36a	29.8 ± 0.69a	34.3 ± 0.74a	11.28 ± 0.39a
N rate (kg ha^− 1^)					
N_240_		20.3 ± 0.27a	27.9 ± 0.70c	36.0 ± 0.60a	9.89 ± 0.38c
N_290_		18.8 ± 0.17b	29.1 ± 0.66b	34.3 ± 0.46b	11.19 ± 0.29b
N_340_		17.1 ± 0.14c	33.2 ± 0.42a	31.1 ± 0.56c	12.18 ± 0.21a
IAA × N rate					
IAA_0_	N_240_	20.1 ± 0.43a	27.0 ± 1.0e	35.8 ± 1.05a	9.70 ± 0.53a
	N_290_	18.5 ± 0.27a	30.0 ± 0.82c	34.3 ± 0.92a	10.90 ± 0.61a
	N_340_	16.9 ± 0.21a	34.0 ± 0.77a	30.0 ± 0.68a	12.40 ± 0.32a
IAA_150_	N_240_	20.4 ± 0.55a	28.3 ± 1.31de	35.2 ± 0.98a	9.83 ± 0.62a
	N_290_	18.9 ± 0.29a	28.0 ± 1.48de	34.5 ± 0.85a	11.27 ± 0.56a
	N_340_	17.1 ± 0.23a	33.8 ± 0.54a	31.2 ± 1.01a	11.87 ± 0.23a
IAA_300_	N_240_	20.5 ± 0.51a	28.3 ± 1.43de	36.8 ± 1.14a	10.15 ± 0.87a
	N_290_	19.2 ± 0.33a	29.2 ± 1.11 cd	34.0 ± 0.77a	11.42 ± 0.35a
	N_340_	17.5 ± 0.26a	31.8 ± 0.54b	32.0 ± 1.13a	12.27 ± 0.51a
*p*-value					
IAA		< 0.001^**^	0.693^ns^	0.027^*^	0.601^ns^
N rate		< 0.001^**^	< 0.001^**^	< 0.001^**^	< 0.001^**^
IAA × N rate		< 0.963^ns^	0.004^**^	0.137^ns^	0.903^ns^
CV (%)		2.8	2.5	2.6	9.5

IAA_0_, IAA_150_ and IAA_300_: spraying of indole‒3‒acetic acid at rates of 0, 150 and 300 mg L^− 1^, respectively; N_240_, N_290_ and N_340_: soil application of nitrogen at rates of 240, 290 and 340 kg N ha^− 1^, respectively. Each value in the table represents mean of three replications ± SE. * and ** indicate significance levels at *p* ˂ 0.05 and *p* ˂ 0.01, respectively; ns denotes no significance. According to Duncan’s multiple range test at *p* ˂ 0.05 different letters indicate significant differences between mean values for each factor in each column. CV: coefficient of variation.

### Juice purity, yield, and nitrogen use efficiency based on root yield (R-NUE)

The IAA level had significant effect (*p* ˂ 0.05) on root yield, pure sugar yield and R-NUE of sugar beet as well as insignificant (*p* > 0.05) effect on loss sugar content and juice purity (Table 5). In this respect, IAA_300_ treatment showed values of root yield, pure sugar yield and R-NUE greater than IAA_0_ and IAA_150_, which reached 1.15 and 1.10, 1.18 and 1.09, and 1.14 and 1.08 times, respectively.

**Table 5:** Loss sucrose content, juice purity, root and pure sugar yields, and nitrogen (N) use efficiency based on root yield (R-NUE) of sugar beet as influenced by indole‒3‒acetic acid (IAA) and nitrogen (N) rate (data average for 2022/23 and 2023/24 cropping seasons)

Treatment		Loss sucrose content	Juice purity	Root yield	Pure sugar yield	*R*-NUE(kg root kg^− 1^*N*)
		(%)		(t ha^− 1^)		
IAA (mg L^− 1^)						
IAA_0_		2.58 ± 0.02a	85.9 ± 0.30a	67.1 ± 2.1c	10.54 ± 0.14c	0.284 ± 0.01c
IAA_150_		2.58 ± 0.03a	86.2 ± 0.34a	70.7 ± 2.4b	11.33 ± 0.20b	0.299 ± 0.01b
IAA_300_		2.59 ± 0.03a	86.3 ± 0.30a	77.2 ± 3.7a	12.50 ± 0.38a	0.324 ± 0.01a
N rate (kg ha^− 1^)						
N_240_		2.57 ± 0.03a	87.3 ± 0.19a	61.1 ± 0.6c	10.81 ± 0.16b	0.321 ± 0.01a
N_290_		2.57 ± 0.02a	86.4 ± 0.21b	67.7 ± 1.0b	11.01 ± 0.18b	0.284 ± 0.01c
N_340_		2.61 ± 0.02a	84.8 ± 0.18c	86.3 ± 2.3a	12.55 ± 0.39a	0.302 ± 0.01b
IAA × N rate						
IAA_0_	N_240_	2.54 ± 0.03a	87.3 ± 0.16a	58.9 ± 0.8 h	10.30 ± 0.21e	0.309 ± 0.01d
	N_290_	2.60 ± 0.04a	85.9 ± 0.28a	65.0 ± 1.0ef	10.32 ± 0.13e	0.273 ± 0.01 g
	N_340_	2.60 ± 0.03a	84.6 ± 0.20a	77.4 ± 2.4c	11.01 ± 0.28de	0.271 ± 0.01 g
IAA_150_	N_240_	2.56 ± 0.06a	87.4 ± 0.42a	61.3 ± 0.9 g	10.91 ± 0.25de	0.322 ± 0.01c
	N_290_	2.54 ± 0.04a	86.5 ± 0.38a	67.0 ± 1.0e	10.94 ± 0.18de	0.281 ± 0.01f
	N_340_	2.63 ± 0.03a	84.6 ± 0.31a	83.9 ± 1.6b	12.13 ± 0.29b	0.294 ± 0.01e
IAA_300_	N_240_	2.62 ± 0.06a	87.2 ± 0.41a	63.0 ± 0.9 fg	11.21 ± 0.26 cd	0.331 ± 0.01b
	N_290_	2.56 ± 0.04a	86.6 ± 0.40a	71.1 ± 1.9d	11.77 ± 0.25bc	0.299 ± 0.01e
	N_340_	2.59 ± 0.04a	85.1 ± 0.38a	97.6 ± 1.6a	14.50 ± 0.36a	0.342 ± 0.01a
*p*-value						
IAA		0.783^ns^	0.138^ns^	< 0.001^**^	< 0.001^**^	< 0.001^**^
N rate		0.106^ns^	< 0.001^**^	< 0.001^**^	< 0.001^**^	< 0.001^**^
IAA × N rate		0.086^ns^	0.171^ns^	< 0.001^**^	< 0.001^**^	< 0.001^**^
CV (%)		1.4	0.5	1.7	3.6	1.3

IAA_0_, IAA_150_ and IAA_300_: spraying of indole‒3‒acetic acid at rates of 0, 150 and 300 mg L^− 1^, respectively; N_240_, N_290_ and N_340_: soil application of nitrogen at rates of 240, 290 and 340 kg N ha^− 1^, respectively. Each value in the table represents mean of three replications ± SE. * and ** indicate significance levels at p ˂ 0.05 and *p* ˂ 0.01, respectively; ns denotes no significance. According to Duncan’s multiple range test at *p* ˂ 0.05 different letters indicate significant differences between mean values for each factor in each column. CV: coefficient of variation.

The N fertilization levels had insignificant (*p* > 0.05) effect on loss sugar content and significant (*p* ˂ 0.05) effects on juice purity, root yield, pure sugar yield and R-NUE of sugar beet. The maximum values of Juice purity and R-NUE were obtained with application of N_240_. While N_340_ was the effective treatment for enhancing root yield and pure sugar yield.

Insignificant (*p* > 0.05) effect on loss sugar content and significant (*p* ˂ 0.05) effects on juice purity, root yield, pure sugar yield and R-NUE of sugar beet were gained due to the interaction between IAA and N rate (Table 5). The highest tested level of both IAA and N (IAA_300_ × N_340_) was the superior practice for improving root yield, pure sugar yield and R-NUE of sugar beet.

### Linear dimensionality reduction (LDR) technique and correlogram for pairwise correlations

**Fig. 2:**
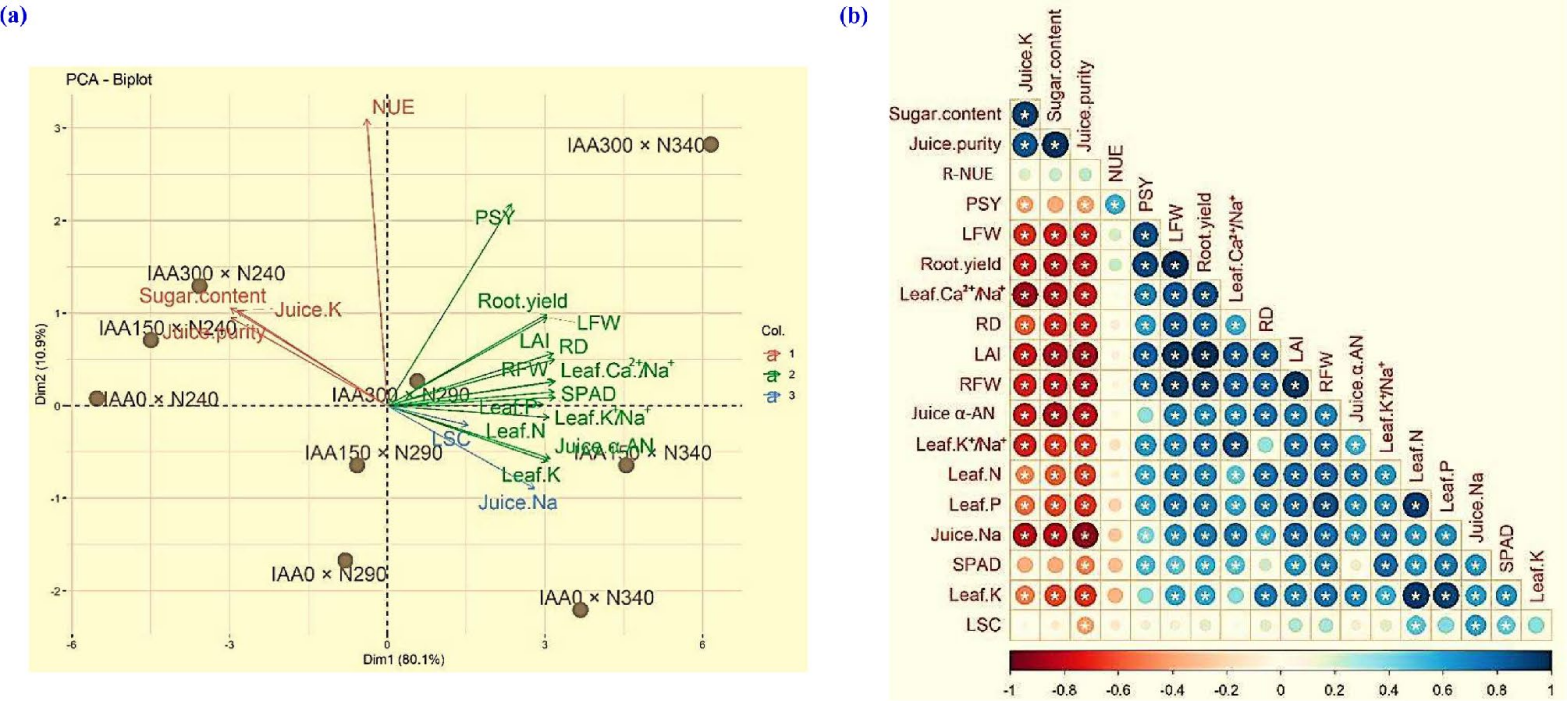
Principal component (PCA) analysis (**a**) for applied indole‒3‒acetic acid (IAA) × nitrogen (N) treatments and studied attributes. Pearson’s correlation coefficients, among the morpho-physiological attributes, yield, and sugar quality-related traits of sugar beet are revealed in the correlogram (**b**). The circle’s size correspond significance level, while * refers to a significant (*p* ˂ 0.05) correlation. IAA_0_, IAA_150_ and IAA_300_: spraying of IAA at rates of 0, 150 and 300 mg L^− 1^, respectively; N_240_, N_290_ and N_340_: soil application of N at rates of 240, 290 and 340 kg N ha^− 1^, respectively. RD: root diameter, RFW and LFW: root and leaf fresh weight, respectively, SPAD: soil plant analysis development, LAI: leaf area index, Leaf N/P/K^+^: leaf nitrogen/phosphorus/potassium, respectively, Ca^2+^ and Na^+^: calcium and sodium, respectively, α-AN: alpha-amino-N, LSC: loss sucrose content, Juice purity, PSY: pure sugar yield, NUE: N use efficiency based on root yield. Each black dot in sub-figure (a) refers to a IAA × N treatment. Values based on average of 2022/23 and 2023/24 cropping seasons

A thorough summary of the relationships among treatments and traits was explored by the principal component analysis (PCA) as a LDR statistical technique which quantified 91% of the variability in the dataset (Fig. 2a). Of the variability 80.1% was explained by the first principal component (Dim1) and 10.9% by the second (Dim2). Sugar-related traits including sugar content, juice purity and juice K were the ones that Dim1 predominantly captured variation in suggesting that they had a major influence on overall treatment distinction. Alternatively, Dim2 explained differences in growth-related traits like SPAD_chlorophyll_, root yield and, and pure sugar yield.

Treatment performance varied significantly according to the PCA dimensions. The treatment IAA_300_ × N_240_ showed potential as the most effective trait to improve sugar quality attributes because of its strong correlation with sugar content, juice purity and juice K. Similarly, IAA_300_ × N_340_ showed a strong correlation with root yield, pure sugar yield and SPAD_chlorophyll_, suggesting that it can be used to increase biomass production and vegetative growth. Other treatments like IAA_0_ × N_240_ and IAA_150_ × N_240_ were situated close to the PCA plots origin showed moderate and non-specialized performance across traits. Juice Na notably showed an inverse relationship with characteristics related to sugar, suggesting that high juice Na levels could have a detrimental effect on juice sugar content and purity. The PCA provided essential insights into treatment selection for desired agricultural productions by clearly illustrating the differences between treatments optimized for biomass output and those optimized for sugar production.

Pearson’s pairwise correlation analysis exhibited significant relationships between sugar beet agronomic traits in our study (Fig. 2b). Root yield showed robust positive correlations with root diameter, root fresh weight, leaf fresh weight, leaf area index, and leaf Ca²⁺/Na⁺ ratio and moderate to strong positive correlations with SPAD_chlorophyll_, leaf N, P, and K^+^/Na^+^ ratio, suggesting that these characteristics positively influence root productivity. Conversely, root yield was negatively associated with sugar content and juice K, indicating potential trade-offs. Pure sugar yield exhibited robust positive associations with root diameter, root fresh weight, leaf fresh weight, leaf area index, leaf N, P, and K^+^/Na^+^ ratio, highlighting its importance in supporting root growth while negatively correlated with sugar content, juice Na, and juice purity. The R-NUE showed moderate positive correlations with pure sugar yield, suggesting that efficient nutrient use is associated with enhanced photosynthetic performance, although weak correlations were found with several leaf nutrient traits.

### Automatic linear modeling (ALM)

**Fig. 3:**
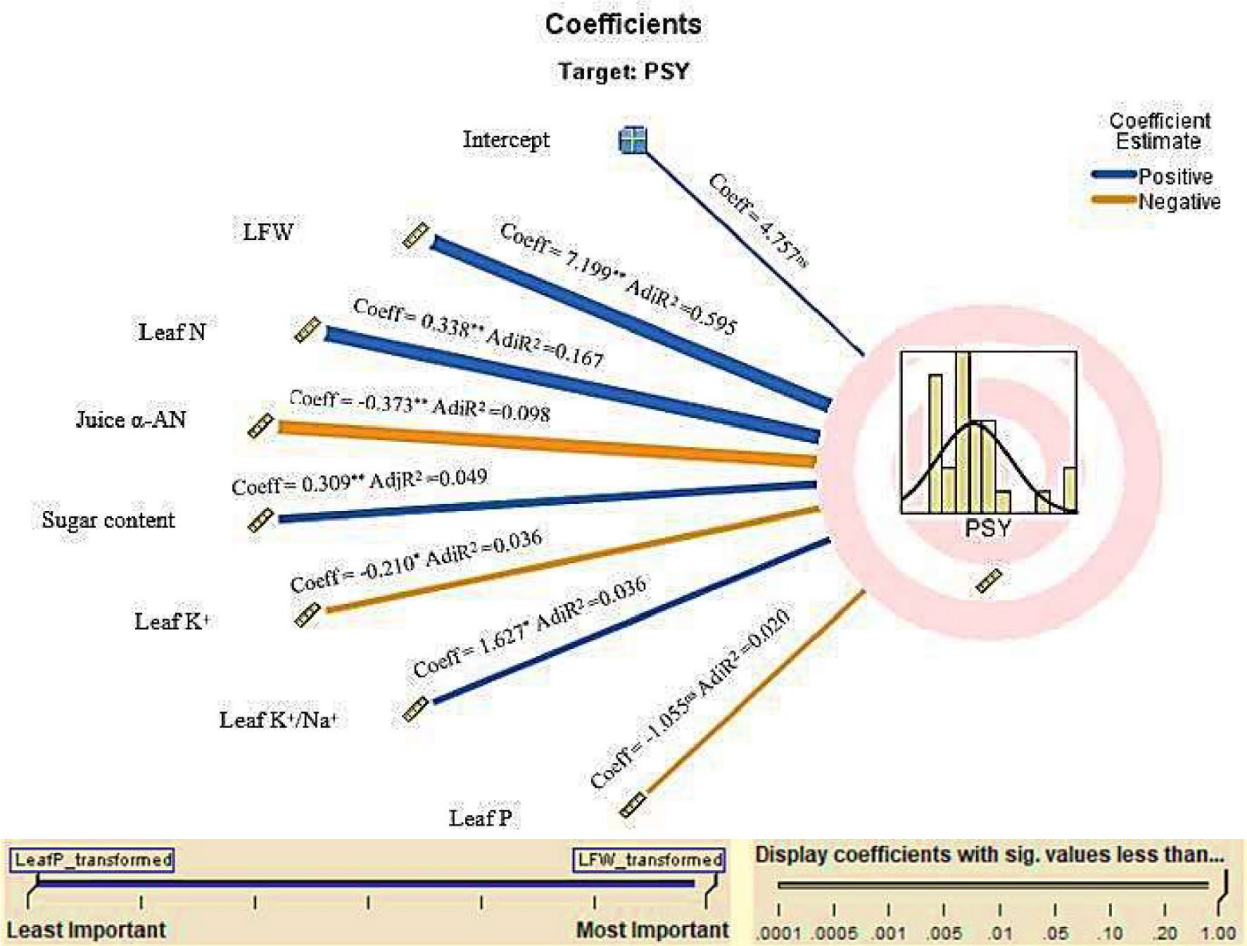
The automated linear modeling diagram of influential attributes (e.g., LFW: leaf fresh weight, leaf N/P/K^+^: leaf nitrogen/phosphorus/potassium, Na^+^: sodium, and α-AN: alpha-amino-N) in sugar beet’s pure sugar yield (PSY) by data of indole‒3‒acetic acid (IAA) × nitrogen (N) interaction (data pooled across 2022/23 and 2023/24 cropping seasons) under saline soil (saturated electrical conductivity = 6.98 dS m^− 1^) conditions. AdjR_2_, Coeff, and sig. stand for adjusted determination coefficient, estimate coefficient, and significance, respectively. * and ** indicate significance levels at *p* ˂ 0.05 and *p* ˂ 0.01, respectively; ns denotes no significance

The IBM SPSS’s ALM is commonly used for automated predictor selection and model fitting. It allows IBM SPSS to evaluate potential variables systematically to select the best-fitting linear regression model. Results indicate that the forward stepwise regression (FSR) model explicates the relationship between pure sugar yield as a target attribute and its explanatory predictors in this study (Fig. 3). Adjusted R^2^ is 0.937 for the outputted FSR model in Fig. 3 denotes that 93.7% of the variations in pure sugar yield are interpreted by variations in leaf fresh weight, leaf N, juice α-AN, sugar content, leaf P, K^+^, and K^+^/Na^+^ ratio (pure sugar yield = 4.757^ns^ + 7.199^**^ leaf fresh weight + 0.338^**^ leaf *N* − 0.373^**^ juice α-AN + 0.309^**^ sugar content − 0.210^*^ leaf K^+^ + 1.627^*^ leaf K^+^/Na^+^ - 1.005^ns^ leaf P). These results highlight the complex interplay of morpho-physio-biochemical traits in determining pure sugar yield, underscoring the critical roles of leaf fresh weight and IAA × N treatment dynamics in optimizing salt-stressed sugar beet productivity under semi-arid conditions.

## Discussion

Notably, the exogenous supply of diversified IAA concentrations enhanced the salt tolerance of sugar beet by improving its physiological and biochemical resilience. Additionally, it mitigated the hazards of salinity by reducing ion toxicity and oxidative stress, thereby protecting plant health. This was clarified well with high concentrations of IAA (300 mg L^− 1^) which increased root diameter, root fresh weight, leaf fresh weight, SPAD_chlorophyll_ value and leaf area index. Promotion of plant growth with alleviation of stress deleterious impact was obtained by IAA application [35–36]. In this respect, in common, abiotic stress and specifically, salinity found to deteriorate the leaf pigments and cause continual atonality in the plant photosystems and reduce gaseous exchange resulting in decreasing plant growth [6, 37–39]. Normally, reactive oxygen species (ROS) are generated with abiotic stresses. Since ROS are malicious molecules, the disintegration rate of vital cell constituents increased [40]. Overaccumulation of ROS occurs under stresses owing to reduction in light absorption and electron transport, causing photooxidation and deactivation in photosystem apparatus [41]. Additionally, exposure to unfavorable conditions during growth stage causes imbalance in plant physiology, hence reduction in yield potential [42–44]. However, plants respond to the stress by accumulating various osmolytes and activating defensive enzymes for ROS scavenging [45, 46]. Enzymatic antioxidants, i.e., catalase, super oxide dismutase and glutathione peroxidase [47, 48], and non-enzymatic antioxidants, i.e., glutathione, phenolic compound, flavonoid, tocopherol, etc. had a strong potential to relieve the concentrations of H_2_O_2_ and O_2_^–^ in stressed plant cell, hence quenching the ROS injuries [49, 50]. As demonstrated in the present study, the application of IAA, particularly at higher concentrations, significantly improved root and leaf growth as well as leaf pigments in salt-stressed sugar beet plants. These findings align with those of Ben Massoud et al. [51], who reported that IAA enhances antioxidant defense mechanisms while mitigating lipid peroxidation damage. However, while our results also indicate an increase in proline accumulation, as noted in previous studies [52], we observed a more pronounced improvement in growth parameters compared to some reports. This variation may be attributed to differences in plant species, salt stress levels, or the concentration and mode of IAA application [53, 54]. Additionally, IAA’s role in promoting ethylene biosynthesis and root elongation, as previously reported [55], was consistent with our findings, though our study further suggests a stronger interaction between IAA and ABA under salinity stress, which may contribute to enhanced root system regulation [56]. Moreover, while IAA has been shown to increase dry weight and total chlorophyll content in various crops [57], the magnitude of these effects in our study suggests that sugar beet may exhibit particularly high sensitivity to auxin under salt stress conditions. These observations underscore the importance of species-specific responses and environmental conditions in shaping the physiological outcomes of IAA application.

Concerning the leaf nutrient content in sugar beet, our results demonstrated the significant role of IAA in enhancing nutrient absorption under salt stress. While previous studies have reported that salt stress disrupts nutrient uptake and metabolism [58–60], our findings align with those of Abdel Latef et al. [36], which also observed increased K^+^, Ca²⁺, and Mg²⁺ ion contents following auxin application. However, unlike Gong et al. [26], which primarily attributed the improvement in N metabolism to enhanced enzymatic activity, our study suggests that IAA-mediated root elongation and capillary root development also play a crucial role in increasing nutrient availability to aerial plant parts. This discrepancy may be due to differences in experimental conditions, including plant species, auxin concentration, and stress severity. Further comparative analysis with studies [12–61] indicates that IAA not only enhances nitrate assimilation but also modulates key enzymes such as nitrate reductase and glutamate dehydrogenase, supporting more efficient N utilization under saline conditions. Thus, our findings provide additional insights into the mechanisms by which auxins mitigate salt-induced nutrient imbalances [62, 63].

In the present study, the application of IAA led to a significant enhancement in CO₂ assimilation, likely due to its role in increasing photosynthetic enzyme activity, which aligns with previous findings [64, 65]. However, while earlier research has demonstrated improvements in root growth, plant pigments, and nutrient content under saline conditions [56], our study further confirms that these effects translate into enhanced root yield and quality traits, particularly under varying salinity levels. Compared to the findings of Qotob et al. [66], who reported improvements in sucrose content and total soluble solids in sugar beet following the foliar application of plant growth regulators, our results indicate that IAA specifically enhances N use efficiency, which may contribute to overall plant performance. The observed differences could be attributed to variations in crop species, experimental conditions, or the specific mode of action of IAA compared to other growth regulators. These findings highlight the potential of IAA in mitigating salinity stress, emphasizing its role in improving physiological and biochemical attributes in stressed plants.

Our results confirm that increasing N fertilizer levels positively influenced sugar beet growth and quality, aligning with previous studies that reported enhancements in chlorophyll content, photosynthetic efficiency, and assimilate translocation [67–69]. In agreement with Wang et al. [69] and Yan et al. [70], we observed that higher N rates improved the photosynthetic rate and dry matter accumulation but led to a decline in R-NUE. However, unlike some previous findings [71–72], where excessive N primarily led to nitrate accumulation in the soil, our results suggest that it also significantly altered nutrient partitioning in sugar beet tissues. Notably, while some studies [73] emphasized that limited N supply inhibits root expansion and reduces sugar content and yield, our findings suggest that moderate N application optimizes sugar yield, increasing it by approximately 46% with a slight improvement (0.2%) in sugar content [74]. Additionally, our results provide new insights into nutrient distribution, as N application enhanced the uptake of Na^+^, K^+^, Ca²⁺, and Mg²⁺, with K⁺ and Ca²⁺ accumulating in the leaves and petioles, while Na⁺ and Mg²⁺ were primarily found in the taproot [70]. These differences in nutrient partitioning might be attributed to variations in soil properties, climate conditions, or differences in sugar beet cultivars used in different studies. Overall, our findings emphasize the importance of optimized N management for maximizing sugar beet productivity while minimizing negative environmental impacts.

It is worth mentioning that the most affected sugar beet traits by the interaction between IAA level and N rate were root diameter, leaf fresh weight, leaf area index, root yield, and pure sugar yield. Previous studies have shown similar results, with amino acids playing a crucial role in regulating plant growth and development, particularly in influencing cell division, differentiation, and acting as antioxidants in the photosynthesis system [75]. However, some differences may arise in the response of plant traits due to variations in experimental conditions, such as the plant species, environmental factors, and nutrient availability. For instance, L-tryptophan, a key metabolite and precursor of auxins like IAA, has been reported to positively influence plant growth by enhancing auxin biosynthesis [76, 77]. The impact of tryptophan on osmolarity regulation, ion transport, and stomatal control has also been noted [78]. In comparison to earlier studies, our findings emphasize a stronger interaction between nitrogen levels and tryptophan in promoting sugar beet growth, which could be attributed to the specific N rate and IAA level combinations tested in our study. The integration of N and amino acids in improving plant growth and productivity has been widely reported, with enhanced nutrient utilization and increased chlorophyll biosynthesis often resulting in better crop yields [79–81]. However, the degree of improvement may vary depending on the concentration and timing of nutrient applications, highlighting the need for further research to optimize these interactions.

Overall, this study highlights the critical role of N levels and IAA interactions in enhancing sugar beet growth and productivity. The findings provide valuable insights into the physio-biochemical mechanisms underlying these effects, particularly the role of amino acids such as tryptophan in auxin biosynthesis and plant stress responses. However, this study has certain limitations, including the focus on a specific range of N and IAA levels, which may not fully capture the broader spectrum of interactions under different environmental conditions. Additionally, while our results demonstrate significant improvements in key agronomic traits, further research is needed to explore the long-term effects of these treatments across multiple growing seasons and soil types. Future studies should also investigate the molecular pathways involved in these interactions to gain a deeper understanding of their regulatory mechanisms. Expanding this research to different crops could further validate the general applicability of these findings in sustainable agricultural practices.

## Conclusions

The synergistic effect of indole‒3‒acetic acid and nitrogen was more pronounced in root diameter, leaf fresh weight, leaf area index, root yield and pure sugar yield of sugar beet. Combination of higher levels of indole‒3‒acetic acid (300 mg L^− 1^) and nitrogen (340 kg N ha^− 1^) showed the distinctive enhancements in most tested traits of sugar beet grown under salt-affected soil. Hence, further research should be performed to test the influence progressive levels, specifically on sugar beet physiology and sugar profile.

## Data Availability

The datasets used and/or analyzed during the current investigation are available from the corresponding author on reasonable request.
